# Association between hospital interval and survival in patients with oral cancer: A waiting time paradox

**DOI:** 10.1371/journal.pone.0224067

**Published:** 2019-10-25

**Authors:** José Luis Lopez-Cedrún, Ana Otero-Rico, Inés Vázquez-Mahía, Juan Seoane, Lucía García-Caballero, Juan Manuel Seoane-Romero, Pablo Varela-Centelles

**Affiliations:** 1 Service of Oral and Maxillofacial Surgery, A Coruña University Hospital (CHUAC), Galician Health Service, A Coruña, Spain; 2 Department of Surgery and Medical-Surgical Specialities, School of Medicine and Dentistry, University of Santiago de Compostela, Santiago de Compostela, A Coruña, Spain; 3 Department of Surgery and Medical-Surgical Specialities, School of Medicine and Health Sciences, University of Oviedo, Oviedo, Spain; 4 Praza do Ferrol Health Centre, EOXI Lugo, Cervo e Monforte de Lemos, Galician Health Service, Lugo, Spain; Chang Gung Memorial Hospital at Linkou, TAIWAN

## Abstract

**Background:**

In early diagnosis studies on symptomatic cancer, survival was the most recommended outcome. The magnitude and impact of the patient interval and primary care interval is well-known in oral cancer; however, the hospital interval and its influence on surviving this neoplasia are not well known.

**Aims:**

To quantify the interval between the first contact with the specialist and the start of treatment for patients with oral cancer and to evaluate whether there was a link between this interval and disease survival.

**Methods:**

We designed a hospital-based study that included 228 patients diagnosed with oral/oropharyngeal squamous cell carcinoma between 1998 and 2008 at A Coruña University Hospital (Spain) who were followed up until 2016. The data were extracted retrospectively from hospital medical charts. The study interval was defined in the context of the "pathways to treatment" model as the interval from the first specialist visit (start point) to the start of treatment (end point). We calculated the total interval (from first symptom to treatment) to evaluate the relative length of the hospital interval, and we considered the variables age, sex, location, comorbidity and tumour classification stage. Survival time was defined as the interval from the first treatment to death or censoring.

**Results:**

The median hospital interval was 20 days, with an interquartile range of 15–29.1 days. The most relevant prognostic variable was the tumour stage (III-IV: Exp. ß = 2.8, p = 0.001). The hospital interval was part of the multivariate model, and its association with mortality showed a V-shaped association, where patients with short hospital intervals (3–18 days) and those with long hospital intervals (26–55 days) had significantly higher mortality than those with medium hospital intervals (19–25 days).

**Conclusion:**

The hospital interval represents a relevant interval for the patient’s path towards treatment, has prognostic implications and is subject to a severity bias (waiting time paradox) that should be avoided.

## Introduction

Oral and oropharyngeal cancer is the sixth most common malignancy worldwide, with an increasing incidence in many European (Czech Republic, United Kingdom, Denmark, Estonia, Finland, Latvia, Norway and Sweden) and Asian countries (Japan and India) [1–3]. Areas with the highest incidence rates include Latin America (Brazil, Uruguay, Puerto Rico and Cuba), South Asia (Sri Lanka, Pakistan and Taiwan) and the Pacific Islands (Papua New Guinea and Melanesia) [1,2].

The estimated annual mortality in 2018 was 177,384 for oral cancer and 51,005 for oropharyngeal cancer, which occurred mainly in Asia and Europe [4]. However, oral cancer continues to be diagnosed mainly in advanced stages (III-IV) [1], which is likely due to diagnostic delays [5], resulting in poor survival (5-year survival of 20–50%). Diagnosing and treating these cancers in the early stages could increase survival by up to 80% [6], thus, preventing delayed diagnoses could be one of the keys to increasing survival in oral cancer.

Although a number of studies have shown inconclusive results when evaluating the association between long periods to diagnosis/treatment and poor outcomes in head and neck cancer [7], various studies have supported a potential association between diagnostic delays and low survival [8].

However, the use of the term "cancer diagnostic delay" has been largely discouraged, given its use outside of a conceptual framework, with heterogeneous criteria that hinder comparisons among studies and provide inconsistent results. To simplify, standardise and monitor studies and interventions aimed at reducing the time to diagnosis and the start of treatment for patients with symptomatic oral cancer, an international Consensus Working Group proposed the "Model of Pathways to Treatment (the Aarhus Statement)”, describing events, processes, intervals and contributing factors from the first symptom (detection of bodily change) to the start of treatment [9,10]. This model has provided up to 15 different intervals to be reported in oncology studies [7] and up to 8 intervals for oral cancer [11,12].

Our study divided the diagnostic/treatment pathway into 2 components: 1) the total pre-hospital interval [13] from the first symptom of oral cancer to when the patient consulted the hospital doctor (patient + primary care interval) and 2) the secondary care interval (from the first consultation in secondary care to treatment) [8–10,13].

Our study focused on the latter hospital interval in the Model of Pathways to Treatment [9,10,13]. Few studies (all outside of a theoretical framework) have considered the hospital delay as a whole in the pathway to diagnosis [14–16] and the start of treatment [17] for patients with oral cancer. A clear association between the magnitude of this time period and tumour staging at the time of diagnosis has also not been established [14–16].

The present study is the first to evaluate the secondary care delay in the hospital setting for oral cancer and the association between hospital time and survival. Our study’s objective was therefore to quantify the interval between the first contact with the specialist and the start of treatment for patients with oral cancer and to evaluate whether there was a link between this interval and disease survival.

## Material and methods

### Patient selection

We designed a hospital-based study that included 231 patients diagnosed with oral/oropharyngeal squamous cell carcinoma between 1998–2008 in the University Hospital A Coruña (Galician Country, Spain). This is one of the two large services of oral and maxillofacial surgery of the Galician Health Service, which serves a population of 2,701,743 people through a free, universal, public scheme. We extracted the data retrospectively from hospital medical charts (paper) by three oral and maxillofacial surgeons (JLL-C, AOR, IVM) directly involved in the diagnosis and treatment of these patients. Patients with pre-hospital histological diagnoses and tumour recurrences and second primary tumours were excluded.

Sample size estimation was based on the principal objective of the study, that was the relation between survival and the interval from the first specialist visit to the start of treatment. The sample size equation selected was based on test for equality for Cox´s Proportional Hazards Model.

Different sample size curves were estimated based on different values for hazard ratios (obtained from different publications with similar objectives) and with different values for overall probability of the occurrence of the event, death by oral cancer (based on regional oral cancer studies). A value of hazard ratio of 1.75 was considered as clinically relevant (in the need for a compromise between the theoretical required sample size and the regional data available). The selected levels for alpha and power (1-beta) were 0.05 and 0.80 respectively.

The required sample size estimated range from 224 (when probability of event is as lower as 45%) to 125 (probability of event of 80%).

The study interval was defined in the context of the "treatment path" model as the interval from the first specialist visit (start point) to the start of treatment (end point), (also known as T14) [7,9,10,18]. We calculated the total interval (from first symptom to treatment: T5) to evaluate the relative length of the hospital interval (T14 / T5). Survival time was defined as the interval from the first treatment to death or censoring.

### Exploratory analysis

The variables age, gender, comorbidity, tumour location, macroscopic pattern and tumour, node and metastasis (TNM) status were considered when estimating the linear regression models. To determine the presence of a relationship between the levels of the variables and patient survival, we adjusted a univariate proportional hazards Cox regression model, including all previously described variables. The model took the following form:

λ(t)=λ0(t)exp{βX},

where X is one of the explanatory variables, λ (t) represents the risk for a given time t and λ0 (t) is the baseline risk.

We then incorporated all the nontemporal explanatory variables into a multiple regression model, which was adjusted with a stepwise regression based on the Akaike information criterion (AIC). We therefore selected the model with the lowest AIC as the best, under the assumption that models presenting multicollinearity are to be rejected. Flexible models were considered for the continuous covariates; however, all covariates behaved linearly and therefore only models with linear effects were considered.

The univariate model whose explanatory variable is the TNM stage did not meet the hypothesis of risk proportionality. We therefore calculated the Kaplan-Meier estimator for the survival function and performed a log-rank test to determine any differences between the various stages.

### Statistical analysis

We employed the mean and median as the central tendency statistics and the interquartile range and 90th centile as the spread indicators when describing the intervals (days). We also calculated the ratio between the mean and the secondary care interval and the total treatment interval (T14 / T5), assuming the conditions for using the test.

To estimate the global survival curve, we employed the Kaplan-Meier method and calculated the estimators associated with the tumour stages (I-II vs. III-IV), applying the log-rank test to identify differences in survival. We then adjusted the multivariate Cox models. The time variable was discretised, as were the non-temporal explanatory variables. We employed the T14 terciles and considered the mean tercile (19–25 days) as the reference level in the adjusted models. All studies were performed using the R software (R Core Team, 2015) [18], with the alpha value indicating significance at the 0.05 level.

The study was conducted according to the Helsinki Declaration guidelines and was approved by the Galician Research Ethics Committee (No. 2014/604), which officially grants patients’ rights and the adequate ethics conditions during research.

## Results

The study’s convenience sample finally consisted of 223 patients, 75% of whom were men (n = 168) and with a median age of 60 years (IQR, 51.7–70.9). The mean time between symptom onset and the start of treatment (total interval T5) was 107.18 days, with a median of 78.0 days (IQR, 55.5–127.5). The mean time between the first visit to specialist care and the start of treatment (hospital setting T14) was 23.4 days, with a median of 20 days (IQR, 15.0–29.1), which represented 25% of the total interval (Table 1). The carcinomas were located mostly on the tongue (33.5%) and floor of the mouth (27.0%), and 54% of the carcinomas were diagnosed in advanced stages. Other, less frequent, locations were gingivae (13.9%), retromolar trigone (11.6%), buccal mucosa (3.7%), and hard palate (0.9%) and were grouped under the heading “others” for analysis. Table 2 lists the sample’s distribution according to the variables considered and the study interval.

**Table 1:** Time intervals. Description of the total time interval (T5) and hospital interval (T14).

Variable	n	Mean	Standard error	Minimum	1^st^ quartile	Median	3^rd^ quartile	Maximum
T5 Interval	183	107.18	85.23	24.00	55.50	78.00	127.50	420.00
T14 Interval	223	23.45	11.16	4.00	15.00	20.00	29.16	63.00
	**Mean (95% CI)**	**Median (95% CI)**						
T14 interval/T5 interval	0.21 (0.10–0.25)	0.25 (0.22–0.28)						

**Table 2:** Sample characterization. Distribution of variables in the sample.

Variable	Absolute frequency	Relative frequency	Mean T14 (days)
**Age**	223		23.453
<40	7	0.031	17.714
40–60	103	0.462	22.680
60–80	99	0.444	24.889
80	14	0.063	21.857
**Gender**	223		23.453
Female	55	0.247	23.145
Male	168	0.753	23.554
**TNM Stage**	223		23.453
I+II	102	0.457	22.814
III+IV	121	0.543	23.992
**N**	220		23.445
N0	145	0.659	24.193
N1	54	0.245	22.093
N2	19	0.086	22.789
N3	2	0.009	12.000
**Tumour site**	215		23.340
Tongue	72	0.335	22.139
Oropharynx	20	0.093	23.200
Floor of the mouth	58	0.270	22.224
Other	65	0.302	25.708
**Comorbidities**	222		23.428
No	146	0.716	23.007
Yes	76	0.284	24.237
**Pattern**	209		23.091
Exophytic	21	0.100	25.575
Ulcerated and mixed	188	0.900	22.814

(T14: Hospital interval).

**Fig 1:**
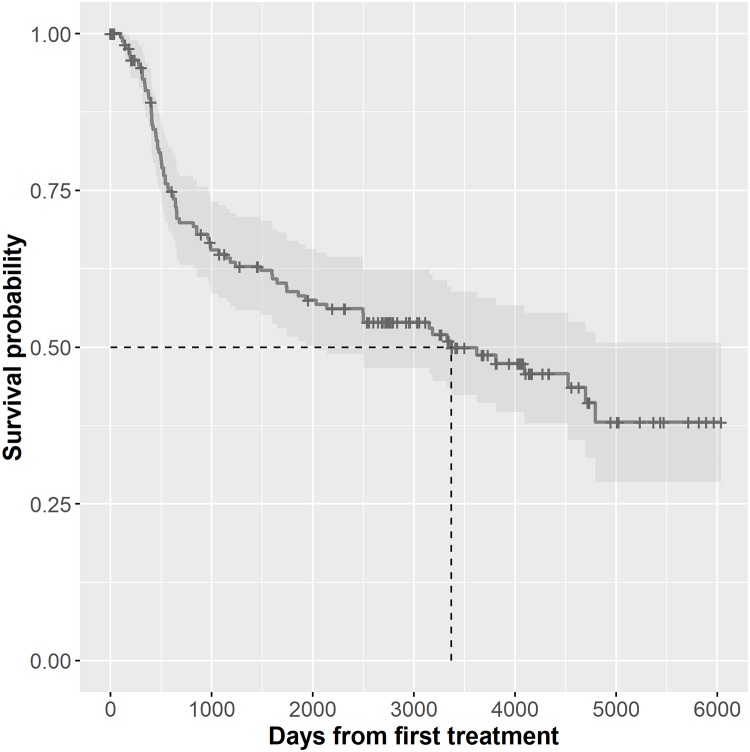
Survival probability. Representation of the Kaplan Meyer curve with the median of the probability of survival.

The median survival was 1953 days, showing a wide distribution range (IQR, 487–3535) (Fig 1). The overall 5-year survival was 35.8% for males and 53.4% for females. The Kaplan-Meier survival curve showed significant differences in survival with regard to the TNM stage (I-II vs. III-IV), and the contrast statistic was 15.6 (p = 0.001).

Univariate Cox Proportional regression models has been estimated for each of the variables included in the final model selected. Variable TNM has been excluded since proportional hazard hypothesis is not met. For each model the estimated coefficients, the hazard ratio, standard error, z-statistic (and corresponding p-value) are presented.

All variables in univariate models (except Sex) are not significant at an alpha level of 0.05. For variable Sex, the risk for men is 1.92 times the risk for women (Table 3).

**Table 3:** Cox survival analysis. Univariate Cox model for patient survival.

	Coefficient (β)	Exp(β)	Std. Error	Z statistic	P-value
**Age**	0.002	1.002	0.0083	0.377	0.706
**Sex: man**	0.6553	1.9258	0.2594	2.526	**0.0115**
**Comorbility: yes**	0.2399	1.2711	0.2005	1.197	0.231
**Tumour site**					
Floor of the mouth	-0.27294	0.76114	0.2652	-1.029	0.303
Oropharynx	-0.29208	0.74671	0.37412	-0.781	0.435
Other	0.03991	1.04072	0.24523	0.163	0.871
T14 (3–18 days)	0.1557	1.1685	0.2453	0.635	0.526
T14 (26–55 days)	0.1758	1.1922	0.2508	0.701	0.483

The multivariate Cox survival model that included the interval (T14) discretised by terciles showed a 2.8-fold greater mortality risk for stages III-IV and a 2-fold greater risk for men. The hospital interval (T14) and mortality show a V-shaped association, where patients with short T14 intervals (3–18 days) and those with long T14 intervals (26–55 days) had higher mortality than those with medium T14 intervals (19–25 days) (Table 4).

**Table 4:** Cox survival analysis. Multivariate Cox model for patient survival.

Variable	Coefficient (ß)	Exp (ß)	Standard error	Z statistic	P-value
Age	0.0074	1.0074	0.0089	0.8230	0.41
Gender: Male	0.6705	1.9552	0.3006	2.2310	0.02*
Comorbidities: Yes	0.0867	1.0906	0.2384	0.3640	0.71
*Tumour site*					
Floor of the mouth	-0.6017	0.5479	0.3139	-1.9170	0.05*
Oropharynx	-0.6129	0.5418	0.4180	-1.4660	0.14
Other	-0.0025	0.9975	0.2749	-0.0090	0.99
TNM: III-IV	1.0334	2.8106	0.2413	4.2830	<0.001*
T14 (3–18) days	0.5651	1.7597	0.2947	1.9180	0.05*
T14 (26–55) days	0.5980	1.8185	0.2918	2.0490	0.04*

(*: statistically significant)

## Discussion

### Limitations and biases

We conducted this study by following the model of pathways to treatment [9,10], adopting the events and intervals generated in the adaptation of the Aarhus guidelines to symptomatic oral cancer [9,11,12]. However, a number of limitations need to be considered.

This was a retrospective, hospital-based study, which could be subject to a selection bias, making it difficult to generalise the results to the general population, but the influence of socioeconomic features is highly unlikely due to the characteristics of the Galician health system. In addition, this type of study has a lower tendency toward information biases (errors in the collected data). In this sense, the memory biases inherent to retrospective studies that could compromise the information recalled by the patients would also affect prospective studies on diagnostic delay. On the other hand, and being the investigation focused on hospital times using clinical records, the chancers for this bias are minimized. However, the fact that researchers involved in the design of the study had also undertaken data retrieval tasks may have resulted in a potential information bias, but the type of data used in our study and the retrospective nature of our investigation makes the existence of this particular systematic error highly improbable.

Our observational study included a large patient sample recruited consecutively with a high inclusion rate (96.5%), making the presence of selection biases unlikely. Eight patients were lost because of the impossibility to retrieve information related to some of the dates defining the interval being studied (hospital interval/T14).

To avoid the presence of confounders (mixing of effects), we considered the exposure variable as the dependent time and adjusted the results of the association according to other prognostic factors (e.g., age, sex, comorbidity, etc.). However, a potential for a classification bias has to be assumed due to the poor discernment between stages II and III in terms of survival inherent to those editions of the AJCC/UICC TNM classifications which do not consider neither depth of invasion (DOI) nor extranodal extension. Tumour aggressiveness could also have been a confounding factor in the association between delay and survival [19]. Calculating the survival interval from the start of treatment instead of from symptom onset could generate a "lead-time bias" (errors in the survival measurement associated with the early detection of cancer) [20,21]. However, this possibility is limited in studies of diagnostic delay in symptomatic oral cancer [20].

The strengths of this investigation include the fact that the features of the simple are similar to the European average for oral cancers, which increases the external validity of the study, the use of a conceptual framework, and the analysis of data about the total time elapsed until treatment. This permits a contextualization of the secondary care in the patients’ path to treatment.

Various systematic reviews have shown an inconsistent relationship (positive association, no association, and even inverse relationship) between diagnostic delay and the risk of recurrence, stage at diagnosis and survival for oral cancer [7,22]. Reports have shown that head and neck cancer (as well as breast, colorectal, testicular cancer and melanoma) has a shorter time to diagnosis, which is associated with better outcomes [7]. A long interval until diagnosis seems to be a moderate risk factor for mortality in head and neck carcinoma [8]. A meta-analysis showed that the probability of presenting an advanced-stage tumour at diagnosis is significantly higher for patients with oral cancer with long intervals to diagnosis than for similar patients with no delay to diagnosis [23].

The total interval in our study resulted to be significantly lower than the average of this time-period calculated from the reports published in the last decade from Australia, India, and Iran [24].

Particularly, the patient interval (patient delay) accounted for the longest period in the patients’ pathway to treatment, although its causes are poorly understood. Some of these factors include denial behaviours, lack knowledge/awareness, self-treatments and physical or economic barriers in the access to care [5,24].

There is extensive knowledge of the intervals associated with patients and primary care [24,25]. However, the secondary care intervals have been scarcely explored. The association between the diagnosis-to-treatment interval (DTI) and survival for oral cancer in the hospital setting has only recently been studied and has yielded conflicting results. Two population-based cancer registry studies have shown poorer survival, with DTIs >20 and >30 days, respectively [26,27]. However, other studies with similar sample sizes have not been able to demonstrate this association for the same time interval [28–30], with DTIs ranging from 22 days [30] to 38 days [28]. The hospital interval also presented wide variability in the literature (15 days to 45 days) [16,17].

In our series, the mean hospital interval was 23.4 days, and patients with short hospital intervals had significantly higher mortality. This counterintuitive association is due to the waiting time paradox (confounding by indication), where seriously ill patients with aggressive tumours (the "sick-quick group") and higher associated mortality are prioritised to prevent the tumour from becoming unresectable or metastasising [31–33]. This phenomenon has also been reported in gliomas, cervical and endometrial cancer, breast cancer [34,35] and colorectal cancer [36]. However, this confounding by severity cannot explain why the longer hospital intervals for oral cancer (>26 days) are significantly associated with higher mortality, suggesting a positive association between long hospital intervals and poorer oral cancer survival rates.

### Clinical implications and future research

The hospital interval is dependent on the characteristics of the clinical practice and the health system and can therefore vary between contexts [37]. Considering the severity bias in the prioritisation of patients with a poorer prognosis for the diagnosis and treatment of oral cancer and that studies have identified that patients undergoing surgical treatment in early stages (I-II) are most affected (in terms of survival) by treatment delay [33–37], strategies should be implemented to promptly treat early-stage patients and prevent stage progression. Given that long hospital intervals generate higher mortality, shortening this interval would increase survival for patients with this neoplasm. Strategies based on multidisciplinary first-day hospital consultations (oral and maxillofacial surgery; ear, nose and throat (ENT); radiotherapy; and medical oncology) have shown the ability to significantly reduce the duration of diagnostic procedures and the delay to the start of the first treatment [38]. Future studies on the early diagnosis of symptomatic oral cancer whose outcome was survival and that followed the Aarhus criteria should control the confounding by indication present in this type of study, thereby establishing the true impact of the intervals to the start of treatment.

Hospital delays represent a significant interval in the patient’s path to treatment. These “delays” have prognostic implications and are susceptible to severity biases (waiting time paradox) that should be prevented.

## Supporting information

S1 File Database used in this investigation. (SAV)
